# Post-transplant outcomes in recipients of living donor kidneys and intended recipients of living donor kidneys

**DOI:** 10.1186/s12882-022-02718-6

**Published:** 2022-03-05

**Authors:** Atit A. Dharia, Michael Huang, Michelle M. Nash, Niki Dacouris, Jeffrey S. Zaltzman, G. V. Ramesh Prasad

**Affiliations:** 1 Division of Nephrology, Department of Medicine, University of Toronto, Toronto, ON Canada; 2 Kidney Transplant Program, St. Michael’s Hospital, 61 Queen Street East, 9th Floor, Toronto, ON M5C 2T2 Canada

**Keywords:** Deceased donor, Graft survival, Living donor, Patient survival

## Abstract

**Background:**

Long-term kidney transplant survival at the population level is consistently favorable, but this survival varies widely at an individual level due to both recipient and donor factors. The distinct contribution of recipient and donor factors to individual post kidney transplant outcome remains unclear. Comparing outcomes in deceased donor (DD) recipients with potential but non-actualized living donors (DD1) to those recipients with actualized living donors (LD), and to DD recipients without potential living donors (DD0) may provide transplant candidates with more information about their own post-transplant prognosis.

**Methods:**

We conducted an observational retrospective cohort study of kidney transplant candidates presenting to our centre for evaluation between 01/01/06 and 31/12/18, and who also received a transplant during that time. Patients were followed to 31/08/2019. Candidates were classified as DD0, DD1, or LD based on whether they had an identified living donor at the time of initial pre-transplant assessment, and if the donor actualized or not. Primary outcome was 5-year death-censored graft survival, adjusted for common pre- and post-transplant donor and recipient risk factors. Secondary outcomes analyzed included patient survival and graft function.

**Results:**

There were 453 kidney transplant recipients (LD = 136, DD1 = 83, DD0 = 234) who received a transplant during the study period. DD0 and DD1 did not differ in key donor organ characteristics. The 5-year death censored graft survival of DD1 was similar to LD (*p* = 0.19). DD0 graft survival was inferior to LD (*p* = 0.005), but also trended inferior to DD1 (*p* = 0.052). By multivariate Cox regression analysis, LD demonstrated similar 5-year graft survival to DD1 (HR for graft loss 0.8 [95% CI 0.25–2.6], *p* = 0.72) but LD graft survival was superior to DD0 (HR 0.34 [0.16–0.72], *p* = 0.005). The 5-year patient survival in DD1 was similar to LD (*p* = 0.26) but was superior to DD0 (*p* = 0.01).

**Conclusions:**

DD recipients with potential but non-actualized living donors exhibit similar mid-term graft and patient survival compared to LD recipients. Having an identified living donor at the time of pre-transplant assessment portends a favorable prognosis for the recipient.

**Supplementary Information:**

The online version contains supplementary material available at 10.1186/s12882-022-02718-6.

## Introduction

Successful kidney transplantation (KT) in patients with end-stage kidney disease (ESKD) improves survival and quality of life compared to chronic dialysis [1].Although registry analyses uniformly report successful graft survival, this survival can often be very difficult to predict for individual patients. This variable post-transplant success motivates ongoing attempts to predict outcomes based on known donor and recipient risk factors, often by using sophisticated models. [2–4] Donor organ source especially affects median graft survival, which is around 19.2 years for living donor transplants and 11.7 years for deceased donor kidney transplants [5]. Living donors have always been considered the ideal kidney source for the recipient, but organs from living donors are not always available.

Donor factors including the organ source are considered variables that are fixed at the time of implantation; post-transplant organ performance then depends on acute perioperative and postoperative immune and load factors [6]. Graft survival depends on how donor and recipient factors interrelate. For example, a kidney of advanced donor age may be more susceptible to ischemic injury, and a donor kidney with acute tubular necrosis may especially depend on good organ perfusion via a healthy recipient cardiovascular system. After transplantation, only the recipient can be actively managed, with the organ source and other donor factors often noted only as unmodifiable determinants of graft success, even though recipient and donor factors continue to interact post-transplant in a relationship situated in the recipient’s environment. However, are there also pre-transplant recipient-donor relationships that continue to affect post-transplant graft survival? Recipient and donor organ identity fuse after transplantation. Outside of registry-level analyses that demonstrate living donor organ superiority, it is common knowledge that many deceased donor transplants individually last longer than some individual living donor transplants. The living donor-deceased donor classification to predict graft survival is thus over-simplified. Donor source is intuitively considered a property exclusive to the organ, but donor source may also be a property of the recipient when graft survival at an individual level varies widely.

If discriminating a living donor from a deceased donor organ source defined at the time of transplantation does not sufficiently predict individual post-transplant graft survival, then examining the intended donor organ source as determined at the time of pre-transplant assessment rather than the actual donor organ source at time of transplantation might add useful information to predicting post-transplant graft survival. Greater explication of the donor source-post-transplant outcome relationship may also help guide public awareness strategies about organ donation, and assist decisions in particular cases about proceeding with living donation or not.

## Material and methods

St. Michael’s Hospital (SMH) is an urban tertiary medical-surgical centre in Toronto, Canada that performs approximately 120 adult single-organ kidney transplants annually and provides post-transplant care to about 1800 kidney transplant recipients (KTR) as of January 1, 2021. KT candidates referred to SMH are assessed by interview and investigations to conform to Canadian guidelines [7]. Whenever transplant candidates are initially referred, their availability of a potential living donor prioritizes the interview date, regardless of the initial likelihood of that donor’s actualization. This policy is meant to facilitate pre-emptive KT and encourage living kidney donation more generally. Potential living donors must self-initiate referral. Living donor availability is then confirmed at recipient interview, and this information is used to guide resource allocation to the remaining workup. If the living donor does not subsequently actualize for any reason, then candidates enter the deceased donor transplant waitlist after establishing their own eligibility provided they had also started dialysis.

In keeping with the current paradigm, we hypothesized that graft outcomes in deceased donor KTR either with or without a previously identified potential living donor will be similar, but the outcomes in both will be inferior to those KTR with actualized living donors. To this end, we performed a retrospective cohort observational study of single-organ KT candidates who were either pre-dialysis or on dialysis and interviewed at SMH between January 1, 2006 and December 31, 2018, and who also subsequently underwent KT at SMH with at least 6 months of post-transplant follow-up. Patients with graft loss within 6 months were included. Data were collected from the SMH transplant program’s electronic database to August 31, 2019. Potential donor source was collected at interview and recorded in the pre-transplant chart, while the ultimate donor source, either living or deceased, was recorded along with demographic variables, early post-transplant events, and post-transplant outcomes from the post-transplant database. KTR with type 1 diabetes mellitus were excluded since they are referred to another centre for the possibility of a subsequent pancreas transplant. Similarly, recipients of a double-kidney or multi-organ transplant were excluded, along with candidates not transplanted during the study period or who were transplanted at another centre.

We compared the graft and patient outcomes of actualized living donor KTR (LD), to both KTR with a prospective living donor but who subsequently received a deceased donor organ because their living donor did not actualize (“intended” LD, or DD1), and KTR without a prospective living donor at initial evaluation who then received a deceased donor organ (DD0). We also compared DD1 and DD0 recipients. This KTR classification was independently verified by two investigators. The primary outcome was 5-year death-censored graft survival, adjusted for donor and recipient age and other characteristics, race, cause of end-stage kidney disease (ESKD), peak panel-reactive antibody (PRA), dialysis duration, acute rejection (AR), and delayed graft function (DGF). Preemptive transplants were assigned a dialysis duration of 0. If a patient received more than one transplant, each transplant was counted as discrete. Secondary outcomes included graft survival at 1 and 3 years, as well as graft function and patient survival at 1-, 3- and 5-years. Graft function based on the estimated glomerular function rate was derived from the CKD-Epi Equation  [8].

Within-group and between-group comparisons were made using paired or unpaired student t-testing, chi-square testing, or Fisher’s exact testing as appropriate. Graft and patient outcomes were compared using Kaplan–Meier methodology and the log-rank test. Independence of predictor variables considered most clinically relevant was verified by multivariable Cox proportional hazards analysis, while avoiding model overfitting. Since this was a population-based study of all KTR transplanted during a pre-defined period, a formal sample size calculation was not performed. Significance was taken as *p* < 0.05. The study was approved by the SMH Research Ethics Board (REB 19–234, October 11, 2019).

## Results

There were 471 KTR who met entry criteria for the study (LD = 152, DD1 = 85, DD0 = 234). A small number of candidates (*N* = 18) were excluded from further study either due to type 1 diabetes mellitus or because they were transplanted at another centre. Reasons for non-actualization of DD1 donors included rejection for medical reasons in 58%, rejection for non-medical (social) reasons in 23%, donor withdrawal in 3%, and loss to follow-up for unknown reasons in 16%. Among all evaluated donors, 46% were biologically related to their recipient, 44% were emotionally related, and 10% had no pre-existing relationship to the recipient (e.g. paired exchange or altruistic donors).

The most common immunosuppressive regimen in recipients was basiliximab induction followed by tacrolimus, mycophenolic acid, and prednisone as maintenance immunosuppression. Table 1 provides baseline demographics. There were 47 preemptive kidney transplants, all in the LD group. There was no difference in the proportion of LD, DD1, and DD0 recipients over the follow-up period (pre-2013 v 2013–18, χ^2^ = 0.41, *p* = 0.57). There were several important differences between the DD0 and DD1 groups, with the DD1 group being younger, on dialysis for a shorter duration, and being more sensitized to human leukocyte antigens (HLA). However, the DD0 and DD1 groups did not differ in important organ characteristics including donor age, terminal serum creatinine; neurological determination of death (NDD) versus donation after cardio-circulatory death (DCD); and standard criteria donor (SCD) versus expanded criteria donor (ECD) status. More DD0 recipients had diabetes and diabetes-related ESKD compared to the other two groups. None of the DD1 and DD0 transplants were preemptive.

**Table 1:** Demographic characteristics of transplant recipients

	**DD0 (*N* = 234)**	**DD1 (*N* = 83)**	**LD (*N* = 152)**	***P* value DD0 v DD1**	***P* value DD0 v LD**	***P* value DD1 v LD**
**Recipient Characteristics**						
**Recipient age at transplant, years mean ± SD (range)**	58.5 ± 11 (22–79)	52.8 ± 13 (24–75)	46.6 ± 13 (17–74)	< 0.001	< 0.0001	0.001
**Male N (%)**	149 (63.7)	53 (63.9)	93 (68.4)	0.976	0.358	0.490
**Race/Ethnicity N (%)**						
Caucasian	82 (35)	32 (39)	87 (57)	0.566	< 0.0001	0.009
Black	42 (18)	11 (13)	7 (5)	0.324	0.0002	0.017
East Asian	55 (24)	14 (17)	25 (17)	0.208	0.094	0.893
South Asian	50 (21)	22 (27)	31 (20)	0.337	0.859	0.311
Hispanic	5 (2)	4 (3)	2 (1)	0.248	0.491	0.148
**Dialysis duration (months)**	70.3 ± 32	60 ± 26	22.1 ± 17	0.012	< 0.0001	< 0.0001
**Peak PRA (%)**	28 ± 33	42 ± 38	16 ± 27	0.004	0.002	< 0.0001
**Smoking N (%)**	55 (23.5)	33 (39.8)	50 (36.8)	0.004	0.006	0.657
**CVD N (%)**	102 (43.6)	34 (40.9)	49 (36.0)	0.677	0.153	0.465
**Cause of End Stage Kidney Disease N (%)**						
Diabetes	65 (28)	14 (17)	24 (16)	0.048	0.011	0.893
Hypertension	19 (8)	5 (6)	8 (5)	0.535	0.280	0.782
Glomerulonephritis	72 (31)	35 (42)	54 (35)	0.059	0.370	0.309
Polycystic kidney disease	18 (8)	10 (12)	23 (15)	0.229	0.019	0.484
Congenital	15 (6)	2 (3)	18 (12)	0.131	0.073	0.010
Interstitial nephritis	6 (2)	6 (7)	7 (4)	0.062	0.249	0.374
Others	39 (17)	11 (13)	18 (12)	0.463	0.201	0.745
**Donor characteristics**						
Donor age, years mean ± SD	49.5 ± 15	48.7 ± 13	42.8 ± 12	0.673	< 0.0001	0.001
NDD/DCD N (%)	171(73)/ 63(27)	63(76)/ 20(24)	NA	0.659	NA	NA
SCD/ECD N (%)	138(59)/ 96(41)	55(66)/ 28(34)	NA	0.295	NA	NA
Terminal serum creatinine (umol/L) mean ± SD	65 ± 22	68 ± 28	NA	0.611	NA	NA
Cold Ischemia Time (minutes) mean ± SD	619 ± 240	622 ± 261	NA	0.23	NA	NA

Two patients received two transplants; each was counted as a discrete transplant. *CVD* Cardiovascular disease, *DCD* Donation after cardiocirculatory death, *ECD* Expanded criteria donor, *NDD* Neurological determination of death, *PRA* Panel reactive antibody, *SCD* Standard criteria donor, *umol/L* micromole/litre. For DD0, DD1, LD definitions see text

**Fig. 1:**
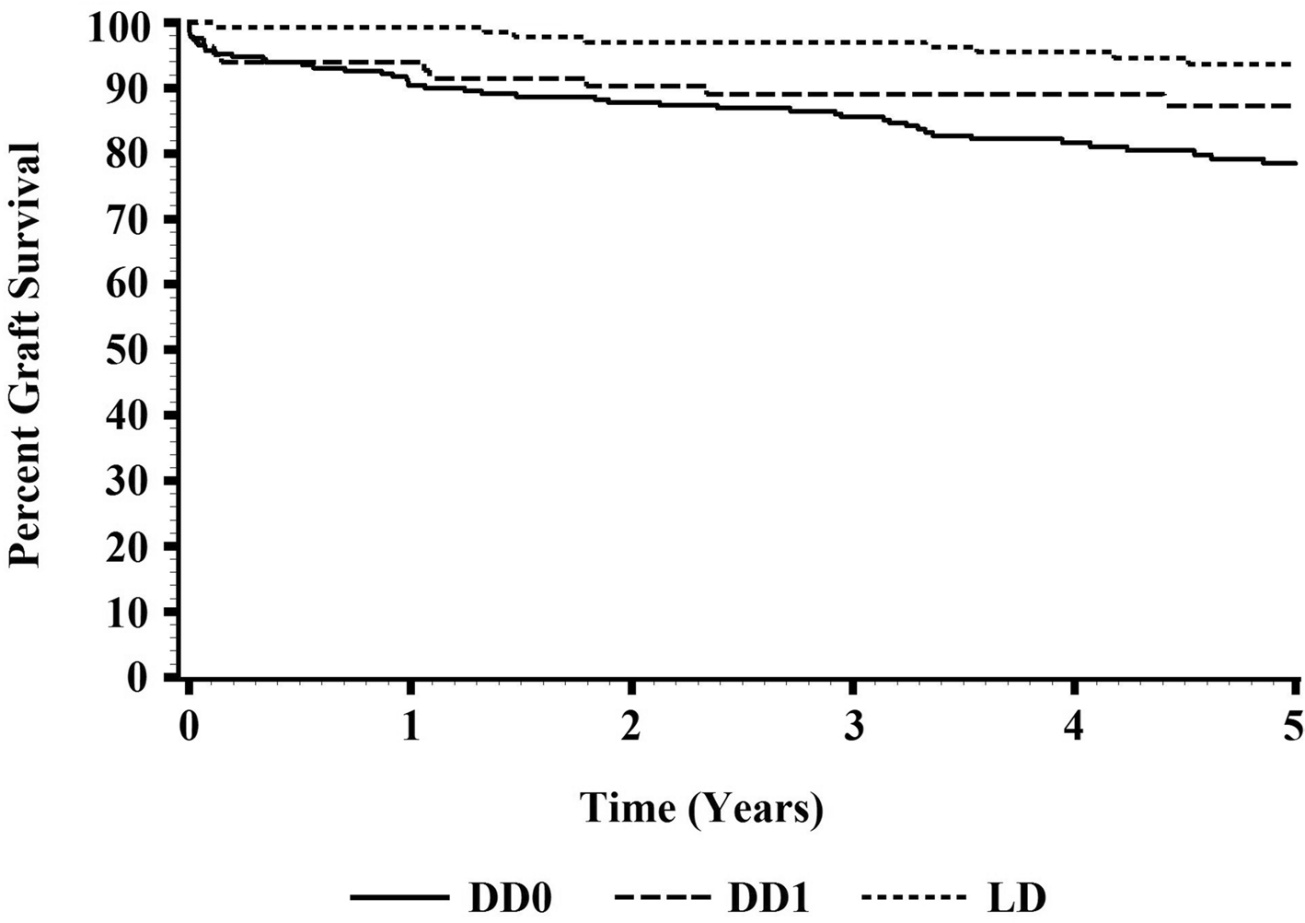
Graft survival in DD recipients without potential living donors (DD0), deceased donor (DD) recipients with potential living donors (DD1), and recipients with actualized living donors (LD)

**Fig. 2:**
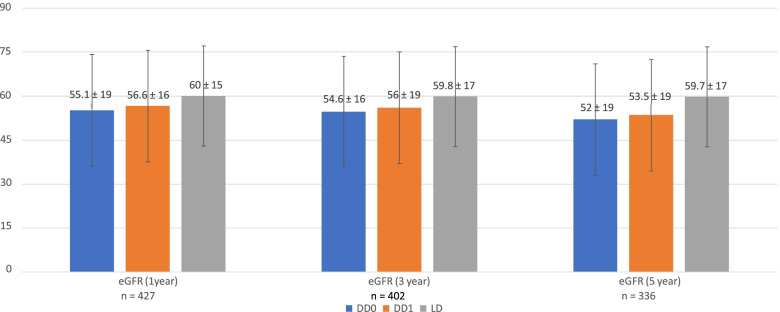
Estimated glomerular filtration rate (eGFR) by the CKD-Epi equation at 1, 3, and 5 years post-transplant in DD recipients without potential living donors (DD0), deceased donor (DD) recipients with potential living donors (DD1), and recipients with actualized living donors (LD). Units are ml/min/1.73m^2^

The 5-year death censored graft survival is shown in Fig. 1, with number of patients at-risk shown in Supplementary Table 1. DD0 trended inferior to DD1 (*p* = 0.052) and was statistically inferior to LD (*p* = 0.005). However, DD1 survival was not inferior to that of LD (*p* = 0.19). LD displayed a higher 5-year eGFR (59.7 ml/min/1.73m2) compared to DD1 (53.5 ml/min/1.73m2) and DD0 (52.0 ml/min/1.73m2) (*p* = 0.009) (Fig. 2). Secondary outcomes are shown in Table 2. The 1 year and 3-year graft survival was higher in the LD group compared to the DD1 and DD0 groups. The 5-year patient survival was superior in the DD1 group compared to the DD0 group. By multivariate Cox regression analysis (Table 3), LD demonstrated superior 5-year graft survival to combined DD (DD0 + DD1) (HR 0.40 [95% CI 0.19–0.84], *p* = 0.016) and DD0 alone (HR 0.34 [0.16–0.72], *p* = 0.005) but not DD1 in isolation (*p* = 0.72). AR, DGF, and diabetic ESKD were also significant predictors of graft survival across models. Adding peak PRA and dialysis duration to each model rendered DD type insignificant (data not shown).

**Table 2:** Secondary outcomes in transplant recipients classified by organ source

	**DD0 (*N* = 234)**	**DD1 (*N* = 83)**	**LD (*N* = 152)**	***P* value DD0 v DD1**	***P* value DD0 v LD**	***P* value DD1 v LD**
DGF N (%)	79 (34)	32 (38)	2 (1)	0.431	< 0.0001	< 0.0001
Acute rejection N (%)	22 (9)	11 (13)	15 (10)	0.323	0.614	0.621
1 year patient survival N (%)	233 (99)	82 (98)	136 (100)	0.195	0.010	0.379
3 years patient survival N (%)	216 (92)	81(97)	133(97)	0.089	0.027	0.628
5 years patient survival N (%)	208 (88)	81 (97)	129 (94)	0.016	0.052	0.268
1 year graft survival N (%)	210 (89)	76 (91)	135 (99)	0.631	< 0.001	0.005
3 years graft survival N (%)	198 (84)	74 (89)	132 (97)	0.308	< 0.001	0.018

Survival is censored for loss to follow-up and death. DGF, delayed graft function. For DD0, DD1, LD definitions see text

**Table 3:** Multivariate Cox regression analysis of factors affecting death censored five-year graft survival

	**Univariate**		**Multivariate**				
**Parameter**	***P* value**	**Hazard ratio (95% confidence interval)**	**Parameter estimate**	**Standard error**	**Chi-square**	***P* value**	**Hazard ratio (95% confidence interval)**
**LD vs DD1**							
LD	0.723	0.812 (0.257—2.566)					
Recipient age (per year)	0.594	1.011 (0.970—1.054)					
Donor age (per year)	0.464	1.014 (0.976—1.054)					
Acute rejection	0.322	1.830 (0.552—6.066)					
Delayed graft function	0.137	2.466 (0.749—8.116)	1.026	0.494	4.310	0.037	2.792 (1.059—7.362)
Caucasian race	0.277	1.730 (0.644—4.651)					
Diabetic Nephropathy as cause for ESKD	0.595	0.692 (0.178—2.694)					
**LD vs DD0**							
LD	0.029	0.408 (0.183 – 0.913)	-1.072	0.385	7.756	0.005	0.342 (0.161—0.728)
Recipient age (per year)	0.140	1.022 (0.993 – 1.051)					
Donor age (per year)	0.822	0.998 (0.979 – 1.017)					
Acute rejection	0.002	2.855 (1.467 – 5.555)	0.967	0.332	8.458	0.003	2.632 (1.371—5.052)
Delayed graft function	0.099	1.633 (0.910 – 2.930)	0.591	0.287	4.237	0.039	1.807 (1.029—3.173)
Caucasian race	0.802	1.076 (0.607 – 1.907)					
Diabetic Nephropathy as cause for ESKD	0.102	1.631 (0.907 – 2.933)	0.579	0.277	4.359	0.036	1.785 (1.036—3.076)
**LD vs DD1 + DD0**							
LD	0.037	0.441 (0.204 – 0.955)	-0.905	0.376	5.795	0.016	0.404 (0.193—0.845)
Recipient age (per year)	0.328	1.012 (0.988 – 1.038)					
Donor age (per year)	0.884	1.001 (0.983 – 1.020)					
Acute rejection	0.001	2.679 (1.482 – 4.844)	0.933	0.296	9.920	0.001	2.542 (1.423 -4.544)
Delayed graft function	0.037	1.742 (1.034 – 2.935)	0.608	0.260	5.439	0.019	1.838 (1.102—3.064)
Caucasian race	0.552	1.169 (0.699 – 1.955)					
Diabetic Nephropathy as cause for ESKD	0.090	1.621 (0.927 – 2.834)	0.548	0.259	4.483	0.034	1.731 (1.042- 2.878)
**DD1 v DD0**							
DD1	0.091	0.555 (0.281—1.097)	-0.698	0.359	3.776	0.052	0.498 (0.246—1.006)
Recipient age (per year)	0.356	1.011 (0.988—1.034)					
Donor age (per year)	0.224	1.011 (0.993—1.029)					
Acute rejection	0.000	3.157 (1.728—5.767)	1.09	0.314	12.053	0.001	2.973 (1.607 – 5.500)
Delayed graft function	0.006	2.049 (1.224—3.429)	0.65	0.274	5.645	0.018	1.916 (1.121—3.275)
Caucasian race	0.870	1.045 (0.615—1.777)					
Diabetic Nephropathy as cause for ESKD	0.010	2.015 (1.185—3.425)	0.645	0.299	4.651	0.031	1.906 (1.061—3.426)

*ESKD* End-stage kidney disease. For DD0, DD1, LD definitions see text

In a post-hoc analysis, we compared the DD1 and DD0 recipients in three additional multivariate models to determine the relative importance of NDD/DCD, SCD/ECD, and cold ischemia time (CIT): these variables plus donor age, terminal serum creatinine and the other variables shown in Table 3; and the variables shown in Table 3 but without post-transplant events including delayed graft function and acute rejection. The results of these models are shown in Table 4.

**Table 4:** Additional Multivariate Cox regression analyses comparing DD1 and DD0

	Univariate		Multivariate				
**Parameter**	***P* value**	**Hazard ratio** **(95% confidence interval)**	**Parameter estimate**	**Standard error**	**Chi-square**	***P* value**	**Hazard ratio (95% confidence interval)**
Model 1							
DD1 (v DD0)	0.090	0.555 (0.281—1.097)					
NDD (v DCD)	0.980	1.008 (0.551—1.843)					
SCD (v ECD)	0.190	1.411 (0.842—2.365)					
CIT (per 60 min)	0.357	1.001 (0.999—1.002)					
Model 2							
DD1 (v DD0)	0.090	0.555 (0.281—1.097)					
NDD (v DCD)	0.980	1.008 (0.551—1.843)					
SCD (v ECD)	0.190	1.411 (0.842—2.365)					
CIT (per 60 min)	0.357	1.001 (0.999—1.002)					
Donor age (per year)	0.223	1.011 (0.993—1.029)					
Terminal serum creatinine (per 10 µmol/L)	0.842	0.999 (0.984—1.013)					
Recipient age (per year)	0.356	1.011 (0.988—1.034)					
Acute rejection	< 0.0001	3.157 (1.728—5.767)	1.636	0.442	13.688	< 0.0001	5.139 (2.159—12.232)
DGF	0.006	2.049 (1.224—3.429)					
Caucasian race	0.869	1.045 (0.615—1.777)					
Diabetic Nephropathy as cause for ESKD	0.009	2.015 (1.185—3.425)					
Model 3							
DD1 (v DD0)	0.090	0.555 (0.281—1.097)					
Recipient age	0.356	1.011 (0.988—1.034)					
Donor age	0.223	1.011 (0.993—1.029)					
Caucasian race	0.869	1.045 (0.615—1.777)					
Diabetic Nephropathy as cause for ESKD	0.009	2.015 (1.185—3.425)	0.690	0.290	5.655	0.017	1.995 (1.129—3.525)

*CIT* Cold ischemia time, *DCD* Donation after cardiocirculatory death, *DGF* Delayed graft function, *ECD* Expanded criteria donor, *ESKD* End-stage kidney disease, *NDD* Neurological determination of death, *SCD* Standard criteria donor, *umol/L* micromole/litre. For DD0, DD1 definitions see text

A small number of DD0 recipients (*N* = 8) subsequently received an LD transplant when a living donor came forward after the initial recipient pre-transplant assessment. However, adding these patients to either the DD0 or LD groups in sensitivity analyses did not significantly affect any results. There was no difference in donor age or recipient age between the DD0 and DD1 groups. There were no DD0 patients known to identify a potential living donor subsequent to their initial assessment (and who could therefore have been assigned to DD1 instead), who then did not actualize.

## Discussion

This study demonstrates important variation in graft survival within deceased donor KTR populations defined at initial evaluation. Graft survival in KTR with a non-actualized living donor did not significantly differ from KTR with an actualized living donor, while both these groups demonstrated outcomes superior to KTR who never had a potential living donor. This difference in graft survival occurred regardless of whether the deceased donor kidney type fulfilled standard criteria or expanded criteria, or occurred from donation after cardio-circulatory or neurological death. These findings suggest that the ultimate source of the donor organ whether living or deceased, while important for graft function and survival, carries with it an important caveat. The heterogeneity in outcome within the deceased donor KTR population likely relates to differences in recipient characteristics, since DD organ quality reassuringly did not differ between the DD0 and DD1 groups. The smaller sample size of DD1 may however have precluded detecting a statistically significant difference between the DD1 and DD0, as well as between the DD1 and LD groups.

KTR with a prospective but non-actualized living donor may share greater similarity to KTR with an actualized living donor than to recipients who never had a prospective living donor at the beginning of their evaluation. The study result therefore raises the interesting hypothesis that potential but non-actualized living donors themselves favorably influence the post-transplant course of their intended recipient. In other words, intended donor source may be a recipient characteristic, while actual donor source is a donor characteristic. It is also possible that candidates with a potential living donor may opt to accept a deceased donor kidney only if considered good quality by the clinical judgment of the clinical team and patient, factors not easily measurable in a retrospective analysis. Medium-term post-transplant outcomes are similar amongst standard criteria deceased and living donor recipients [9]. A risk prediction score combining functional, histological and immunological parameters had good discrimination ability to predict long-term graft loss. When those parameters were assessed at time of transplantation, none were independently associated with long term graft survival [10].

Differences in DD recipient outcomes may reflect social support, medication adherence, and other recipient characteristics. While important biological factors pertaining to the organ influence post-transplant outcome, the present study shows that post-transplant outcomes differ among deceased donor recipient groups despite equivalent donor biological quality. Therefore, non-biological factors may be just as important. For example, a previous study demonstrated that longer-term graft survival is worse in recipients of living donor kidneys obtained through transplant tourism compared to domestic deceased donor transplants, and this inferiority of living donor transplants could not be explained by early post-transplant events [11]. Therefore, recipient factors may be more important than donor factors in longer-term graft survival, while donor factors are more important in shorter-term graft survival. More provocatively, donor organ source may be considered a recipient factor affecting graft survival.

The simple binary discriminatory variable of having a potential living donor identified many years before the actual transplant takes place may be a useful tool to predict medium-term graft survival. Large registry studies do not provide the required level of data granularity for pre-transplant information. Outcomes are typically captured based on actualized donors, living or deceased. Similarly, a randomized control trial of actualizing or non-actualizing a potential living donor is neither practical nor ethical. Studies to capture sociological variables are intense to perform and limited to small patient numbers. Granular single-centre level data about potential living donors on the other hand may be the best source for information that links social characteristics to graft survival.

Predictors of living donor dropout may include female recipient, systemic disease as cause of kidney failure in the recipient, or relationship as a friend [12]. Social factors have been investigated before as predictors of post-transplant kidney graft function and survival. Higher self-efficacy score, younger age and higher income have all been associated with having a potential living donor. Self-efficacy, defined as a person’s belief that he or she is capable of accomplishing a particular goal helps find a living donor [13]. Self-efficacy is important to self-management [14]. Besides self-efficacy, post-transplant medication adherence is vital to transplant success, and an important risk factor for medication non-adherence is poor social support [15]. Receiving a living donor kidney also associates with greater social participation [16]. It is possible that many of the social benefits of receiving a living donor organ pertain also to recipients who had an intended living donor, especially if the intended donor continues to be a component of the post-transplant environment. Social science research is however painstaking, and not feasible on a large scale to many transplant programs. Moreover, the relationship of social support to adherence and post-transplant outcomes more generally is inconsistent and remains unclear [17]. Efforts to develop self-management scales can be cumbersome. Recording the presence or absence of an identified potential living donor at the time of initial post-transplant assessment on the other hand cuts across many social variables, and can be a simple method to help differentiate candidates to their future risks regarding post-transplant outcomes, without needing to be a statistically independent variable. Not having an identified living donor at the time of the initial pre-transplant assessment can then be marked in the post-transplant chart as a potential risk factor for inferior graft outcome. Such recipients can then be more efficiently targeted to more intense post-transplant attention and intervention across both biological and social domains. These measures may not be as necessary for deceased donor KTR who had a potential but non-actualized living donor.

The present study has several strengths and limitations. To our knowledge, this is the first study to identify the potentiality, not just actuality of a living kidney donor as a predictor variable for post-KT graft survival. The study splits the deceased donor recipient population into two sub-populations, who may be at different risk for adverse post-transplant events. The study also highlights the critical importance of transferring pre-transplant social variables to the post-transplant record. The variable is simple and inexpensive to collect, and can be routinely collected by large registries in the future. The study is limited by its single-centre and retrospective nature, and must therefore be considered hypothesis-generating. We were unable to collect detailed information on donor-recipient relationships beyond biological relationship, reasons for donor drop-out, or post-transplant social factors. Since the number of events was small, we could not dissect their nature further or control for more than only a limited number of potentially confounding variables. We did not have information on human leukocyte antigen mismatch or pre-transplant donor specific antibodies. Future studies can help elucidate social factors and the effect of corresponding interventions, but in the meantime reproducing these findings at other transplant centres and through registries is needed. While larger sample sizes and longer follow-up will be valuable to confirming these findings, there remains sufficient reason for transplant centres to henceforth collect this simple binary pre-transplant variable. KT candidates whose living donors do not actualize may also obtain some reassurance that their post-transplant outcomes will not necessarily be inferior to LD transplant recipients if they accept a DD organ. Since the potential living donor’s organ still lives outside the recipient’s bodily confines, an identified potential living donor may be serving as a surrogate for numerous impossible-to-collect post-transplant recipient factors affecting graft survival.

## Conclusions

Deceased donor kidney transplant recipients with a potential but not actualized living donor demonstrate mid-term graft survival comparable to living donor kidney transplant recipients. Deceased donor transplant recipients without a potential living donor demonstrate outcomes inferior to both these groups, and may benefit from more targeted study of assessments and interventions to improve their post-transplant graft survival.

## Supplementary Information

**Additional file 1.**

## Data Availability

The datasets generated and/or analysed during the current study are not publicly available due to limitations of ethical approval involving the patient data and anonymity but are available from the corresponding author on reasonable request.

## Abbreviations

AR: Acute rejection
DCD: Donation after cardiocirculatory death
DD: Deceased donor
DGF: Delayed graft function
ECD: Expanded criteria donor
ESKD: End-stage kidney disease
KT: Kidney transplantation
KTR: Kidney transplant recipient
LD: Living donor
NDD: Neurological determination of death
PRA: Panel-reactive antibody
SMH: Saint Michael’s hospital
SCD: Standard criteria donor
