# Supplementary material for: Post-transplant outcomes in recipients of living donor kidneys and intended recipients of living donor kidneys
Source: BMC Nephrol. 2022 Mar 5;23:97. doi: 10.1186/s12882-022-02718-6 (PMC8898413; doi:10.1186/s12882-022-02718-6)
Supplement: Supplementary file 1 — Additional file 1. [file 12882_2022_2718_MOESM1_ESM.docx]

**Supplementary Table 1**

Number of patients at risk for graft loss in Figure 1

|  | Number of At-Risk Patients | | | | | |
| --- | --- | --- | --- | --- | --- | --- |
|  | Year | | | | | |
| Group | 0 | 1 | 2 | 3 | 4 | 5 |
| DD0 | 234 | 206 | 200 | 193 | 139 | 113 |
| DD1 | 83 | 77 | 74 | 73 | 59 | 49 |
| LD | 152 | 134 | 130 | 129 | 113 | 90 |

For DD0, DD1, LD definitions see text.
